# The perfect storm: Disruptions to institutional delivery care arising from the COVID-19 pandemic in Nepal

**DOI:** 10.7189/jogh.11.05010

**Published:** 2021-05-11

**Authors:** KC Ashish, Stefan Swartling Peterson, Rejina Gurung, Alkistis Skalkidou, Jageshwar Gautam, Honey Malla, Punya Paudel, Kumari Bhattarai, Nisha Joshi, Bhim Singh Tinkari, Shree Adhikari, Durgalaxmi Shrestha, Binda Ghimire, Seema Sharma, Laxmi Khanal, Sunil Shrestha, Wendy Jane Graham, Mary Kinney

**Affiliations:** 1 Department of Women’s and Children’s Health, Uppsala University, Sweden; 2 Department of Global Public Health, Karolinska Institute, Stockholm, Sweden; 3 Research Division, Golden Community, Lalitpur, Nepal; 4 Ministry of Health and Population, Kathmandu, Nepal; 5 Family Welfare Division, Department of Health Services, Nepal; 6 Paropakar Maternity and Women’s Hospital, Nepal; 7 Bheri Hospital, Nepalgunj, Nepal; 8 Nepal Nursing Council, Kathmandu, Nepal; 9 Seti Provincial Hospital, Dhangadi, Nepal; 10 White Ribbon Alliance Nepal, Kathmandu; 11 London School of Hygiene & Tropical Medicine, London, UK; 12 School of Public Health, Faculty of Community and Health Sciences, University of the Western Cape, Cape Town, South Africa

## Abstract

**Background:**

The COVID-19 pandemic has led to system-wide disruption of health services globally. We assessed the effect of the pandemic on the disruption of institutional delivery care in Nepal.

**Methods:**

We conducted a prospective cohort study among 52 356 women in nine hospitals to assess the disruption of institutional delivery care during the pandemic (comparing March to August in 2019 with the same months in 2020). We also conducted a nested follow up cohort study with 2022 women during the pandemic to assess their provision and experience of respectful care. We used linear regression models to assess the association between provision and experience of care with volume of hospital births and women’s residence in a COVID-19 hotspot area.

**Results:**

The mean institutional births during the pandemic across the nine hospitals was 24 563, an average decrease of 11.6% (*P* < 0.0001) in comparison to the same time-period in 2019. The institutional birth in high-medium volume hospitals declined on average by 20.8% (*P* < 0.0001) during the pandemic, whereas in low-volume hospital institutional birth increased on average by 7.9% (*P* = 0.001). Maternity services halted for a mean of 4.3 days during the pandemic and there was a redeployment staff to COVID-19 dedicated care. Respectful provision of care was better in hospitals with low-volume birth (β = 0.446, *P* < 0.0001) in comparison to high-medium-volume hospitals. There was a positive association between women’s residence in a COVID-19 hotspot area and respectful experience of care (β = 0.076, *P* = 0.001).

**Conclusions:**

The COVID-19 pandemic has had differential effects on maternity services with changes varying by the volume of births per hospital with smaller volume facilities doing better. More research is needed to investigate the effects of the pandemic on where women give birth and their provision and experience of respectful maternity care to inform a “building-back-better” approach in post-pandemic period.

The COVID-19 pandemic has placed unprecedented pressure on health systems globally. Preparedness and response for COVID-19 case management have acutely overwhelmed and strained routine health services, including maternity services [1,2]. with some devastating results. The early expectation that COVID-19 would have an indirect impact on maternal, newborn and child health services [3] has now been backed by mounting evidence that the pandemic has indeed led to the disruption of essential health services for women and their children during pregnancy, childbirth and postnatal period [4], with some devastating results [1,5-8].

Both the supply of and demand for maternity care have been impacted. Health facilities have undergone preparation and reorganization of their maternity services in order to triage and handle the COVID-19 cases [9]. Maternity health care workers have experienced extraordinary challenges, including lack of personal protective equipment, ever-changing or unclear clinical guidelines, harassment and violence, and overall increased workloads due to reallocation of staff to COVID-19 dedicated areas or self-isolation due to exposure or illness [10,11]. On the demand side, the COVID-19 era has also witnessed changes in care seeking behaviour [5-8,12], with reports of delayed or reduced utilization of maternity services [5,13]. These delays result in adverse birth outcomes [14], and are compounded by issues of inequity [15]. The COVID-19 pandemic has exposed the health system fragility and has widened the equity gap for the most vulnerable population [16].

Maternity care and the progress made in maternal and newborn health (MNH) in the last 25 years are under threat due to the COVID-19 pandemic [3,17]. This progress includes health outcomes as well as a woman’s right to high quality, dignified, respectful care [18,19], as protected in global human rights law and policy [20,21] and laid out in the Respectful Maternity Care (RMC) Charter [22] and as recommended by the World Health Organization (WHO) [23,24]. Experience from other health crises, such as the Ebola outbreaks in West Africa, showed that quality and respectful care diminished and led to declines in facility deliveries [25-27]. During the current pandemic, there has been widespread concern and some reporting about violations of RMC, such as unnecessary separation of women and their newborns, or denial of care [1,4,28]. The problem is exacerbated by health worker shortages and other health system strains as well as patient fear of exposure to the infection at the health facility [4,29-31].

Our recent study from Nepal reports on the devastating impact of the national COVID-19 lockdown on maternity services, including increased risk of stillbirth and neonatal mortality as well as reduced facility births, increased risk of complications, and issues around the provision of quality care [15]. Prior to the COVID-19 pandemic, Nepal had been making remarkable strides to reduce maternal and perinatal mortality, yet there were still growing concerns of overcrowding in the higher-level referral facilities, under-utilization of the primary level referral facilities, and gaps in referral services between levels [32,33]. The global evidence of poor quality maternity care during the COVID-19 pandemic raises concern that there could also be an increase in disrespect and abuse in Nepal, despite policies protecting a woman’s right to RMC [34].

To investigate this concern, we set out to understand the disruption of maternity services in Nepal due to the COVID-19 pandemic as well as to explore the provision and experience of institutional delivery care. We hypothesize that the arrival of COVID-19 created a perfect storm [35], wherein the disruptions to the health system, notably institutional maternity care, combined with existing challenges of referrals and sub-optimal utilization of services, created an unusual combination of events and circumstances resulting in reduced quality of maternity care (Figure S1 in the **Online Supplementary Document**).

## METHODS

### Design

We conducted a prospective cohort study in nine hospitals in Nepal where larger quality improvement studies, REFINE [36] and SUSTAIN [37] have been implemented since 2019. Our study assessed two components: 1) disruption in maternity services, and 2) respectful maternity care, defined as the provision and experience of institutional delivery care. For the first part, we compared the monthly trends in institutional births and bed occupancy rates in postnatal care units between two time periods, March-August 2019 and March-August 2020 (during the pandemic). There were 52 356 women enrolled (27 856 for March-August 2019 and 24 500 for March-August 2020) and who consented to the REFINE and SUSTAIN studies. For the second component of the study, we conducted a nested cohort observational study as well as follow up interviews at 45 days with 2022 women who had a live birth at the hospitals during the pandemic to assess their provision and experience of institutional delivery care (Figure S2 in the **Online Supplementary Document**).

### Settings

Seven of the hospitals were secondary referral (provincial) level hospitals while two were primary referral (district) hospitals (Dadeldura and Surkhet). All hospitals provide Comprehensive and Emergency Obstetric and Neonatal Care services (CEmONC). The nine hospitals, distributed across all seven provinces, covered 11 · 2% of the national number of births for 2019 [36,37]. From March-August 2020, the Government of Nepal reported 39 459 COVID-19 cases with wide variations across the provinces (Figures S3-6 in the **Online Supplementary Document**) and with a national lockdown imposed for this period.

### Study participants

For the disruption of maternity services, we included all the women-infant pair consented and enrolled to REFINE [36] and SUSTAIN [37] studies. Women at 22 weeks of gestation or more admitted in the labour room with fetal heart sound at admission were eligible for this study. For the nested cohort study and follow up interviews, to measure the uptake and experience of care, a subset of women (10%) who were part of the REFINE and SUSTAIN studies with live births (April 11-July 5, 2020) born either vaginally or caesarean births were enrolled and followed up at 45 days postpartum with a phone interview. To use the individual level data for this study, an additional approval was taken from the ethical review board of Nepal Health Research Council (reg No. 439/2020).

### Patient and public involvement

This study aimed to assess the disruption of maternity services and respectful childbirth care during COVID-19. Since there is a need to redesign the health system so that there is a continuity of care during pandemics and disease outbreaks, the study was conducted together with health workers, health managers and policy makers.

### Data collection

Data on institutional births was collected from the hospital record registry. The number of health workers in the labour and delivery room and those who were redeployed to COVID-19 area, and the number of days’ maternity service were disrupted during the pandemic were assessed through observation from independent research nurses. The socio-demographic and provision of care data were extracted from the existing data collection system for REFINE and SUSTAIN studies. For these studies, a validated clinical observation checklist was used to observe the labour and delivery event for all vaginal births, and women’s obstetric and neonatal information was collected from inpatient case notes. A data collection system was set up at each hospital, and independent clinical researchers did observations using a tablet-based application. To assess women’s experience of care, a validated semi-structured questionnaire was used for follow up interviews with women by telephone at 45 days postpartum to assess their experience of care during childbirth [38].

### Measurement

To assess the first component of the study, disruption of maternity services, we measured the institutional births before the pandemic (March-August 2019) and during pandemic (March-August 2020) by hospital. For each period, we measured the number of health workers in the labour and delivery room and the postnatal care bed occupancy rate (BOR) [39]. Specific to the period during the pandemic, we considered if hospitals had a dedicated COVID-19 space in the labour and delivery ward (ie, separate room) as well as if the hospital had reported any days of service disruption, ie, no maternity services provided for these days.

For the second component of the study, we considered respectful maternity care as the quality of maternity care during childbirth, considering both the provision and experience of care. To measure provision of care, we considered six components of the health worker’s performance during intrapartum care based on WHO’s 2016 “Standards for improving quality of maternal and newborn care in health facilities [23].”: 1) health worker’s hand washing practice before childbirth was defined as health care staff who did clean their hands correctly as per the WHO’s five moments for hand hygiene, 2) health worker using gloves and gown to protect from infection transmission during childbirth, 3) preparation of equipment for birth, 4) health workers greet the mother at the time of admission, 5) newborn kept skin-to-skin contact with mother after birth, and 6) newborn breastfed within one hour of birth. To measure the experience of care, we considered six components from the woman´s perspective based on the abuse and disrespect typology by Browser and Hill:[40] 1) verbal or physical abuse, 2) treated with dignity as measured by the health workers’ sensitive handling of the perineal area, 3) delay or carelessness during the birth, 4) women’s experience of care on any verbal or physical abuse, by health workers, 5) effective communication-a) health worker’s informed and took consent before performing vaginal examination and b) women informed and consented before caesarean birth and 6) supportive care to women a) women counselled on keeping the baby warm, b) women counselled on exclusive breast feeding and c) women counselled on newborn danger signs.

To assess the presence of a “COVID hotspot”, women were asked at admission if they knew of any case of COVID-19 within the same community as their residence in the last seven days. If they indicated yes, then the participant was recorded as coming from a COVID hotspot area. None of the women were screened for COVID-19 infection upon admission during the pandemic period.

For socio-demographic characteristics, women’s ethnicity was assessed based on the caste system [41] in Nepal (ie, relatively disadvantaged ethnic groups (Janajati, Madeshi, other disadvantaged, Dalit) and relatively advantaged ethnic groups (Brahmin and Chhetri-Hill, and Brahmin-Tarai). Women’ age was assessed as a mean with standard deviation in each group and categorised as 18 years or younger, 19-24 years, 25-29 years, 30-34 years, and 35 years or older. Parity was measured as women who had no previous birth, at least one previous birth, or two or more previous births. Obstetric characteristics measures included: mode of birth including spontaneous vaginal birth, assisted vaginal birth and caesarean birth. For neonatal characteristics, we captured preterm birth less than 37 weeks of gestation based on first day of last menstrual period and sex of baby as boy or girl or ambiguous.

### Data analysis

Hospitals were categorized into four groups based on the average number of daily deliveries during 2019 (Table S1 in the **Online Supplementary Document**): Group 1, low-volume delivery (1-8 births per day); Group 2, medium-volume delivery (9-16 births per day); Group 3, high-medium volume delivery (17-24 births per day); Group 4, high-volume delivery (25-33 births per day). To assess the disruption of maternity services, we calculated the average rate of change between the two time periods for the following measures: the institutional births and BOR. We used ANOVA test to assess the difference in institutional birth and BOR between two time periods. We calculated the mean difference for the number of health workers in the labour room and the number of days that the maternity services were disrupted. BOR was calculated as the number of postnatal beds occupied divided by the total number of postnatal beds available in the hospital multiplied by the number of days in the hospital (include time period), with the ratio multiplied by 100 [39].

The coverage of provision and experience of institutional delivery care were analysed among the cohort of women-infant pair for only the pandemic period. The differential factors affecting the provision of care were analysed based on residence in a COVID-19 hotspot area, the volume of deliveries per day, and complications during birth. The factors affecting experience of care were analysed by women’s ethnicity, age category, parity, complications during admission, mode of birth, prematurity at birth, sex of child and the volume of deliveries per daily.

We created two continuous score indices, one for the respectful provision of care and one for the respectful experience of care, based on the above indicators, using principal component analysis (PCA). PCA is a dimension reduction technique used for combining many variables into a single one. The usual practice is to weight the variable according to the first principal component ie, the component that has the highest variance, and thus the highest discriminatory power. The provision and experience of care variables with coverage of 95% or less were included. The first principal component for respectful provision of care index had 40.5% variance, the highest variance, and was considered to measure the care. The first principal component for respectful experience of care index had 42.5% variance, highest variance, and was considered to the measure of care. The index score ranged from -2 to +2, with former indicating poor provision or experience of care while the latter indicating good provision or respectful care.

We conducted bi-variate and multivariate linear regression to assess the association of respectful provision of care index and respectful experience of care index with four parameters: place of residence of women in relation to COVID-19 hotspots, socio-demographic characteristics, prematurity and volume of births per day. By analysing the data within these four parameters using the linear regression, we were able to assess the independent interaction of the social and obstetric characteristics with two separate indices. We used STATA 17.0 to manage and analyse the data (Stata Corp, College Station, TX, USA).

## RESULTS

### Disruption of maternity services

The mean number of institutional births in March-August 2019, 27793, decreased by 11.6% to 24563 (*P* < 0.0001) in the corresponding months of 2020. The average rate of change in institutional births between these two periods varied greatly by hospitals (**Table 1**). Six hospitals had a decline in births (range 10.5% to 26.1%); Pokhara had the greatest reduction. Three hospitals had an increase in monthly births (range 1.9% to 23.7%); Dadeldhura Hospital, a low-volume hospital, had the greatest increase in monthly births (23.7%, *P* < 0.0001) whereas Seti Provincial Hospital, a high-medium volume hospital, declined by 26.6% (*P* = 0.001) during the pandemic period. The average decline in the monthly births varied by the hospital group (volume of births) with the greatest reduction in Group 3 (high-medium volume hospitals) at 20.8% (*P* < 0.001) and Group 2 (low-medium-volume hospitals) at 19.4% (*P* < 0.001) during the pandemic but less declines in Group 4 (high-volume hospitals) at 4.3% (*P* = 0.033); and Group 1 (low-volume hospitals) at 7.9% (*P* = 0.001) (Table S2 in the **Online Supplementary Document**).

**Table 1:** Disruption of maternity care services in the 9 hospitals of Nepal

	Group, based on average number of births per day*	Mean Institutional births before pandemic	Mean Institutional births during pandemic	Institutional births -change before-after the pandemic (*P*-value)	Number of HW^†^ before pandemic	Number of HW during pandemic	BOR in PNC unit^‡^ before pandemic	BOR in PNC unit during pandemic	BOR change before- after the pandemic, *P*-value	COVID-19 dedicated care in labour room	Number of days service disrupted^§^
Overall		27793	24563	-11.6%, (<0.0001)	6	5	91.0% (75.2, 106.8)	81.8% (65.6, 97.9)	0.421	Yes	4.3
Dadeldhura hospital	Group 1, low volume	582	720	23.7%, (<0.0001)	4	4	40.6% (29.7, 51.4)	79.2% (69.5, 88.9)	<0.0001	Yes	0
Surkhet Provincial	Group 1, low volume	2047	2116	3.4%, 0.064	4	4	68.3% (50.4, 86.1)	68.6% (56.5, 80.6)	0.976	Yes	0
Bheri Provincial	Group 2, medium volume	2225	1900	-14.6%, (<0.0001)	6	6	48.7% (37.8, 59.7)	45.2% (35.1, 55.4)	0.651	Yes	3
Seti Provincial hospital	Group 2, medium volume	2963	2189	-26.1%, (<0.0001)	6	5	43.7% (36.4, 51.0)	25.9% (22.9, 28.9)	0.001	Yes	10
Koshi hospital	Group 2, medium volume	3228	2691	-16.6%, (<0.0001)	8	6	69.4% (51.9, 86.9)	52.3% (40.2, 64.3)	0.141	Yes	8
Janakapur provincial	Group 3, high medium volume	3111	2613	-16.0%, (<0.0001)	6	6	117.1% (100.9, 133.3)	42.7% (27.6, 57.8)	<0.0001	Yes	3
Pokhara	Group 3, high medium volume	3414	2554	-25.2% (<0.0001)	6	4	79.4% (60.4, 98.5)	57.6% (42.6, 72.6)	-0.104	Yes	6
Bharatpur	Group 4, high volume	5175	4634	-10.5%, (0.032)	8	8	196.1% (144.4, 247.7)	182.7% (155.5, 209.9)	0.66	Yes	6
Lumbini provincial	Group 4, high volume	5048	5146	1.9%, (0.453)	8	6	155.4% (122.7, 188.1)	181.6% (143.2, 220.0)	0.327	Yes	3

HW – health worker, BOR – bed occupancy rate, PNC – postnatal care

*Group 1 (1-8 births per day); Group 2 (9-16 births per day); Group 3 (17-24 births per day); Group 4 (25-33 births per day).

†Number of health workers per 24 h in the labour room.

‡Bed occupancy rate in the postnatal unit.

§Number of days that maternity services were not provided.

The average BOR across the nine facilities in their postnatal units declined from 91.0% (95% CI; 75.2, 106.8) before the pandemic to 81.8% (95% CI = 65.6, 97.9) during the pandemic (*P* = 0.421). Again, Dadeldhura had the greatest BOR increase (40.6% to 79.2%, *P* < 0.0001), and Seti had the second greatest decrease in BOR (43.7% to 25.9%, *P* = 0.001), Janakpur had the overall greatest BOR decrease (117.1% to 42.7%, *P* < 0.001). The BOR for Group 3 (high-medium volume) hospitals had the greatest decrease during the pandemic (98.3% to 50.1%, *P* < 0.0001) and Group 4 (high-volume) hospitals reported over 100% capacity (Table S3 in the **Online Supplementary Document**). All of the hospitals reported a dedicated room in the labour and delivery ward for COVID-19.

Before the pandemic, the number of health workers per 24 hours in the labour room ranged from four to eight across the hospitals with an average of 6.2 health workers. This staffing level decreased to 5.4 health workers during the pandemic period due to the redeployment of staff to COVID-19 dedicated care. Three hospitals had a reduction of two health workers during the pandemic (Koshi, Pokhara and Lumbini) but no changes took place in the Group 1 hospitals (low-volume). Maternity services were halted for an average of 4.3 days during the pandemic because of a disease outbreak in the labour room (range from 0 to 10 days) over the period March-August 2020. Seti hospital reported the largest number of days disrupted, 10 days, but no disruptions at all were reported in Dadeldhura and Surkhet (**Table 1**).

### Respectful provision of care during childbirth during COVID-19

Table 2 presents the coverage of indicators measuring the provision of care. Health workers washed hands before attending to a delivery in 52.0% of the births. The coverage was higher for deliveries among women who arrived from a COVID-19 hotspot area than those from non-hotspot areas (60.2% vs 41.9%). The coverage of handwashing before childbirth was highest in the lower volume hospitals and lowest in the highest volume hospitals. Preparation of equipment for immediate newborn care occurred in 86.4% of births, with higher coverage in the lower volume hospitals. Across all hospitals, 33.6% of women were greeted upon admission, again with higher coverage among women from lower volume facilities. The coverage of women greeted upon admission was higher among women who arrived from a COVID-19 hotspot area than those from non-hotspot areas (44.8% vs 23.3%).

**Table 2:** Coverage of provision of care and co-variate variance

	Health workers washes hand before childbirth	Health workers uses gloves on both hand and gown	Preparation of equipment for immediate newborn care	Health workers greet mothers	Baby kept skin to skin contact	Breast feeding initiated inside labour room
Overall coverage of care	52.0% (48.9, 55.1)	97.8% (96.7, 98.6)	86.4% (84.1, 88.4)	33.6% (30.7, 36.6)	21.5% (19.0, 24.2)	48.4% (45.3, 51.6)
**COVID-19 area:**
Yes	60.2 (55.5, 64.8)	94.1 (91.3, 96.0)	85.2 (81.5, 88.3)	44.8 (40.1, 49.6)	21.2 (17.4, 25.6)	44.8 (39.9, 49.8)
No	41.9 (38.0, 45.9)	90.9 (88.3, 93.0)	77.8 (74.3, 80.9)	23.3 (20.0, 26.8)	21.5 (18.3, 25.2)	49.7 (45.5, 53.9)
**Number of daily births:**
Group 1, 1-8 births	77.1 (69.5, 83.2)	95.8 (91.0, 98.1)*	96.5 (91.9, 98.6)	56.9 (48.7, 64.8)	14.9 (9.9, 21.8)	48.9 (40.8, 57.2)
Group 2, 9-16 births	50.8 (46.4, 55.3)	94.2 (91.8, 96.0)*	88.7 (85.5, 91.2)	37.7 (33.5, 42.1)	19.1 (15.7, 23.0)	46.7 (42.2, 51.3)
Group 3, 17-24 births	40.1 (34.8, 45.7)	89.2 (85.2, 92.2)*	69.8 (64.4, 74.6)	19.8 (15.7, 24.5)	27.4 (22.4, 33.0)	52.2 (46.3, 58.1)
Group 4, 25-33 births	33.3 (25.5, 42.3)	89.2 (82.2, 93.6)*	61.7 (52.7, 69.9)	11.7 (7.0, 18.6)	25.5 (18.1, 34.6)	45.3 (36.1, 54.8)
**No complication during admission:**
No	49.9 (46.8, 53.0)	93.2 (91.4, 94.6)	81.8 (79.2, 84.1)	32.5 (29.6, 35.5)	20.7 (18.2, 23.5)	48.4 (45.2, 51.7)
Yes	41.5 (31.3, 52.4)	82.9 (73.2, 89.6)	73.2 (62.6, 81.6)	26.8 (18.4, 37.4)	31.8 (21.7, 43.9)	48.5 (36.7, 60.4)

*Statistically significant.

**Figure 1:**
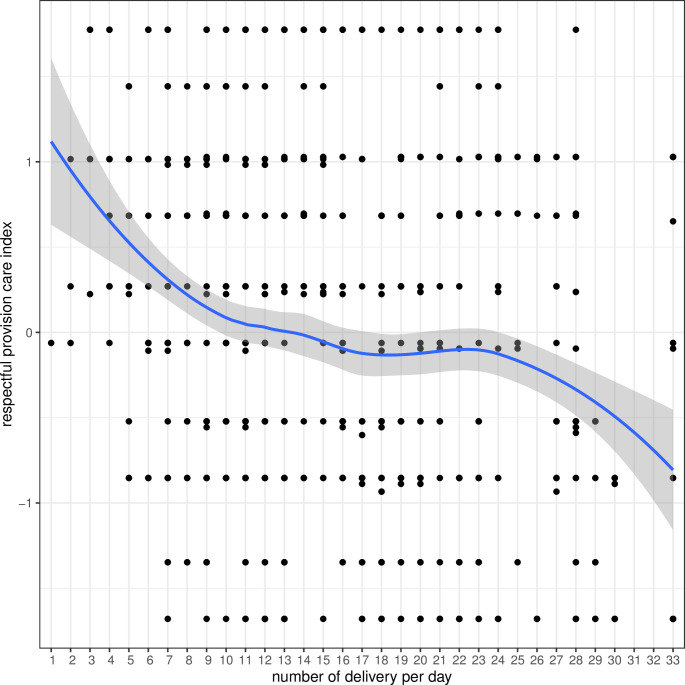
Respectful provision of care by volume of birth.

In a bi-variate analysis of the associations with the respectful provision of care index, shown using box plots, respectful provision of care was greater among women arriving from COVID hotspot areas than those who did not arrive from a COVID hotspot area. A higher respectful provision of care score was found among the multi-parous women than first time mothers (Figure S6 in the **Online Supplementary Document**). There was also a higher score for the respectful provision of care among preterm babies and for Group 1 hospitals (low-volume) (Figure S7 in the **Online Supplementary Document**). With each increase in the average number of institutional births per day, there was a decline in the respectful provision of care score (**Figure 1**).

Using the multi-variate linear regression analysis, there was an increase in respectful provision of care with women coming from a COVID hotspot area (β = 0.275, *P* < 0.0001) in reference to women not coming from a COVID hotspot area. The respectful provision of care was better with number of daily births limited to Group 1 (low-volume) hospitals (β = 0.446, *P* < 0.0001) in reference to Group 3 (high-medium volume) hospitals. The respectful provision of care was less in Group 5 (high-volume) hospitals (β = -0.335, *P* = 0.002) in reference to Group 3 (high-medium volume) hospitals (**Table 3**).

**Table 3:** Bi-variate and multi-variate linear regression on the association between co-variates and respectful provision care index during COVID-19 period

	Bi-variate analysis			Multi-variate analysis			
	**β estimate**	**t-value**	***P*-value**	**β estimate**	**t-value**	***P*-value**
Global intercept				-0.247	-4.042	0
Women's arrival from COVID-19 hotspot						
Intercept	-0.132	-2.996	0.003			
No	**Reference**			**Reference**		
Yes	0.308	4.636	<0.0001	0.275	4.172	<0.0001*
Volume of births per day in the hospital:
Intercept	0.048	0.993	0.321			
Group 1, 1-8 births per day	0.391	3.982	<0.0001	0.446	3.903	<0.0001*
Group 2, 9-16 births per day	**Reference**			0.087	1.127	0.26
Group 3, 17-24 births per day	-0.172	-2.228	0.026	**Reference**		
Group 4, 25-33 births per day	-0.488	-4.498	<0.0001	-0.335	-3.149	0.002*
Complication during admission:
Intercept	-0.007	-0.202	0.84			
No	**Reference**			**Reference**		
Yes	0.103	0.773	0.439	0.147	1.133	0.258

*Statistically significant.

### Respectful experience of care during COVID-19

Table 4 presents the coverage of indicators measuring the experience of care. The vast majority of women reported no verbal or physical abuse (95.4%) and no delay or carelessness (91.4%). No one reported discrimination during childbirth and most women were informed and consented for caesarean birth (95.6%). Counselling indicators had lower coverage, with 40.0% of women reporting they were counselled on keeping the newborn warm, 44.1% of women counselled on exclusive breastfeeding and 15.3% of women counselled on newborn danger signs. Consent before performing vaginal examination was higher among primiparous women than nulliparous women (55.3% vs 44.3%). The counselling to women on keeping the baby warm was higher among multi-parous women than first time women (47.9% vs 36.3%). Group 1 hospitals (low-volume) had a higher proportion of women who were consented for vaginal examination than the other groups (**Table 4**).

**Table 4:** Coverage of experience of care and co-variate variance

	Consent for PV	Informed before emergency CS	No delay or carelessness or ignored during the care	Counselling on keeping the baby warm	Counselling on exclusive breast feeding	Counselling on newborn danger signs
Overall coverage of care	50.2% (48.1, 52.4)	95.6% (93.5, 97.1)	91.4% (90.1, 92.6)	40.0% (37.9, 42.2)	44.1% (42.0, 46.3)	15.3% (13.8, 16.9)
Ethnicity:
Dalit, relatively disadvantaged	49.8 (43.5, 56.1)	93.9 (82.6, 98.0)	91.6 (87.3, 94.5)	35.4 (29.6, 41.7)	43.5 (37.3, 49.9)	15.2 (11.2, 20.4)
Janajati, relatively disadvantaged	48.3 (44.3, 52.3)	98.2 (94.4, 99.4)	91.4 (88.9, 93.4)	37.4 (33.6, 41.3)	41.9 (38.0, 45.9)	11.5 (9.1, 14.3)
Madhesi, relatively disadvantaged	46.8 (42.1, 51.5)	89.4 (80.9, 94.4)	89.8 (86.6, 92.3)	35.2 (30.8, 39.8)	44.9 (40.3, 49.6)	15.5 (12.4, 19.2)
Muslim, relatively disadvantaged	38.8 (29.1, 49.5)	84.2 (60.8, 94.8)	90.6 (82.3, 95.2)	40.0 (30.2, 50.7)	47.1 (36.7, 57.7)	12.9 (7.3, 21.9)
Brahmin/Chhetri, relatively advantaged	55.9 (52.1, 59.7)	98.3 (95.0, 99.5)	92.5 (90.2, 94.3)	47.2 (43.3, 51.0)	45.0 (41.2, 48.8)	18.6 (15.8, 21.8)
Other, relatively advantaged	52.0 (33.1, 70.4)	90.0 (53.2, 98.6)	96.0 (76.4, 99.4)	44.0 (26.3, 63.4)	56.0 (36.6, 73.7)	24.0 (11.2, 44.2)
Age category (years):
15-19	48.7 (40.8, 56.6)	82.8 (64.6, 92.7)	92.8 (87.4, 96.0)	33.6 (26.5, 41.4)	40.1 (32.6, 48.1)	11.8 (7.6, 18.0)
20-24	49.0 (45.8, 52.2)	95.3 (91.4, 97.4)	92.5 (90.6, 94.0)	39.6 (36.5, 42.7)	46.8 (43.6, 50.0)	15.9 (13.7, 18.4)
25-29	51.6 (47.8, 55.5)	98.3 (94.9, 99.5)	89.8 (87.2, 91.9)	38.7 (35.0, 42.5)	41.2 (37.4, 45.0)	13.2 (10.7, 16.0)
30-34	53.1 (46.6, 59.6)	95.8 (87.7, 98.6)	91.5 (87.1, 94.5)	47.8 (41.3, 54.3)	45.1 (38.7, 51.7)	18.3 (13.8, 23.9)
≥35	48.4 (36.3, 60.7)	94.1 (67.9, 99.2)	88.7 (78.2, 94.5)	48.4 (36.3, 60.7)	40.3 (28.9, 52.9)	25.8 (16.5, 38.1)
Parity:
No previous birth	44.3 (40.9, 47.8)	93.9 (90.0, 96.4)	91.8 (89.6, 93.5)	36.3 (33.0, 38.8)	43.5 (40.1, 47.0)	12.9 (10.8, 15.4)
1 previous birth	55.3 (51.7, 58.9)	98.8 (95.3, 99.7)	91.2 (88.9, 93.1)	38.6 (35.1, 42.1)	42.1 (38.6, 45.7)	14.0 (11.7, 16.7)
2 or more previous birth	52.3 (47.9, 56.6)	94.4 (88.2, 97.5)	91.2 (88.5, 93.4)	47.9 (43.6, 52.3)	47.9 (43.6, 52.3)	20.9 (17.5, 24.7)
Complication during admission:
No	49.6 (47.3, 51.9)	95.0 (92.3, 96.7)	91.1 (89.6, 92.3)	39.5 (37.3, 41.8)	43.6 (41.3, 45.9)	15.2 (13.6, 17.0)
Yes	55.3 (48.9, 61.6)	98.1 (92.8, 99.5)	94.5 (90.7, 96.8)	43.8 (37.6, 50.2)	48.1 (41.8, 54.5)	15.7 (11.6, 21.0)
Mode of delivery:
Spontaneous vaginal delivery	45.5 (43.0, 48.1)	NA	90.9 (89.4, 92.3)	38.6 (36.2, 41.2)	43.5 (41.0, 46.1)	15.1 (13.4, 17.0)
Instrumental delivery	50.0 (37.6, 62.4)	NA	95.0 (85.6, 98.4)	46.7 (34.5, 59.2)	41.7 (29.9, 54.4)	13.3 (6.8, 24.5)
Caesarean birth	64.0 (59.7, 68.0)	95.6 (93.5, 97.1)	92.5 (89.8, 94.5)	43.2 (38.9, 47.5)	46.1 (41.8, 50.5)	16.0 (13.1, 19.5)
Sex:
Boy	51.5 (48.6, 54.5)	95.8 (92.7, 97.6)	92.2 (90.5, 93.6)	42.6 (39.7, 45.5)	45.1 (42.2, 48.0)	16.4 (14.3, 18.7)
Girl	48.7 (45.5, 52.0)	95.4 (91.7, 97.5)	90.6 (88.5, 92.3)	36.9 (33.8, 40.1)	43.0 (40.0, 46.2)	13.8 (11.7, 16.2)
Ambiguous	0	100	0	0	0	100
COVID-19 hotspot:
No	50.5 (47.6, 53.4)	96.2 (93.3, 97.9)	91.8 (90.1, 93.3)	38.4 (35.6, 41.2)	44.7 (41.9, 47.6)	15.1 (13.1, 17.2)
Yes	50.1 (46.5, 53.6)	94.8 (90.5, 97.2)	91.3 (89.1, 93.1)	41.1 (37.7, 44.6)	41.8 (38.3, 45.3)	15.8 (13.4, 18.6)
Number of daily births:
Group 1, 1-8 births	63.4 (58.0, 68.5)	98.6 (90.6, 99.8)	93.9 (90.7, 96.0)	60.9 (55.5, 66.1)	62.2 (56.8, 67.3)	28.6 (24.0, 33.8)
Group 2, 9-16 births	48.4 (45.0, 51.8)	93.1 (88.7, 95.9)	89.7 (87.4, 91.6)	37.2 (33.9, 40.5)	42.6 (39.3, 46.1)	15.6 (13.3, 18.2)
Group 3, 17-24 births	47.0 (43.1, 50.9)	97.2 (93.3, 98.8)	92.4 (90.1, 94.2)	33.4 (29.8, 37.2)	40.0 (36.3, 43.9)	9.5 (7.4, 12.0)
Group 4, 25-33 births	47.5 (41.3, 53.8)	96.4 (86.6, 99.1)	91.8 (87.6, 94.7)	38.9 (33.0, 45.2)	35.7 (29.9, 41.9)	11.5 (8.0, 16.1)

PV – per vaginal examination, CS – caesarean section

**Figure 2:**
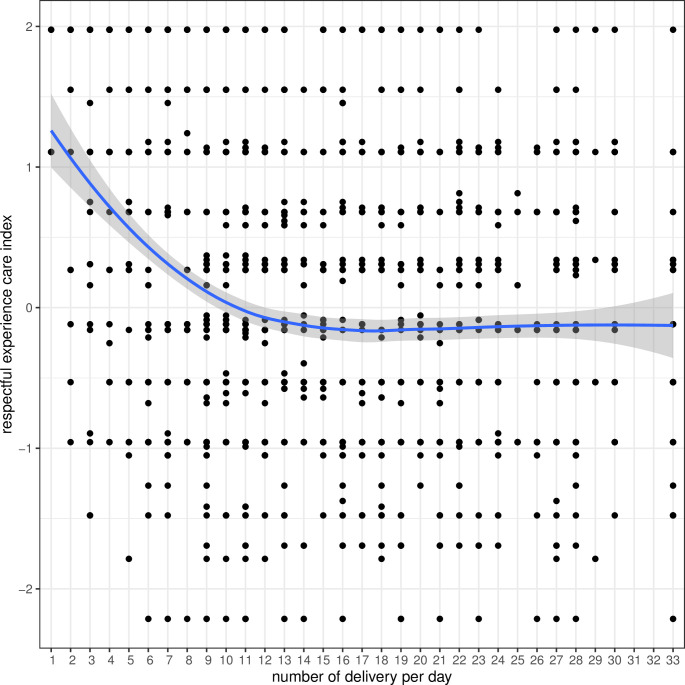
Respectful experience of care by volume of birth.

In a bi-variate analysis on associations with the respectful experience of care index, the score was highest in Group 1 (low-volume) hospitals and lowest in Group 3 (high-medium volume) hospitals (Figure S9 in the **Online Supplementary Document**). With each increase in the average number of institutional births per day, there was a decline in the respectful experience of care score (**Figure 2**).

Using multi-variate linear regression analysis, there was an increase in respectful experience of care for women coming from a hotspot area (β = 0.076, *P* = 0.001) in reference to women not coming from a hotspot area. Women from Janjati, a relatively disadvantaged ethnic group, had an inverse association with the respectful experience of care (β = -0.184, *P* = 0.001). Women who had two or more previous births reported better experience of respectful care (β = 0.175, *P* = 0.003) compared to those with no previous birth. Women who had a caesarean birth reported better care (β = 0.194, *P* < 0.0001) as compared to women who had a spontaneous vaginal birth. Women with preterm birth reported more respectful care (β = 0.188; *P* = 0.002) in reference with term birth. The respectful experience of care was better in Group 1 (low-volume) hospitals (β = 0.549, *P* < 0.0001) in reference to Group 3 (high-medium volume) hospitals (**Table 5**).

**Table 5:** Bi-variate and multi-variate linear regression on the association between co-variates and respectful experience care index during COVID-19 period

	Bi-variate linear regression			Multi-variate linear regression		
	**β coefficient**	**t-value**	***P*-value**	**β coefficient**	**t-value**	***P*-value**
Global intercept				-0.039	-0.599	0.549
Ethnicity:						
Intercept	0.125	3.206	0.001			
Dalit	-0.162	-2.143	0.032	-0.164	-2.159	0.031
Janajati	-0.215	-3.799	<0.0001	-0.184	-3.273	0.001*
Madhesi	-0.173	-2.79	0.005	-0.221	-3.392	0.001*
Muslim	-0.176	-1.534	0.125	-0.282	-2.398	0.017
Brahmin/Chhetri	**Reference**			**Reference**		
Others	0.085	0.418	0.676	-0.064	-0.322	0.747
Parity:						
Intercept	-0.084	-2.371	0.018			
No previous birth	**Reference**			**Reference**		
1 previous birth	0.07	1.377	0.169	0.005	0.09	0.928
2 or more previous birth	0.236	4.147	<0.0001	0.175	2.94	0.003*
Mode of birth:						
Intercept	-0.045	-1.712	0.087			
Spontaneous vaginal birth	**Reference**			**Reference**		
Assistant vaginal birth	0.066	0.505	0.614	0.062	0.473	0.636
Caesarean birth	0.171	3.325	0.001	0.194	3.774	<0.0001*
Preterm birth:						
Intercept	-0.035	-1.441	0.15			
No	**Reference**			**Reference**		
Yes	0.205	3.483	0.001	0.188	3.13	0.002*
COVID-19 hotspot area:						
Intercept	-0.006	-0.212	0.832			
No	**Reference**			**Reference**		
Yes	-0.004	-0.09	0.928	-0.042	-0.943	0.346
Number of daily births:						
Intercept	-0.056	-1.652	0.099			
Group 1, 1-8 births per day	0.573	8.984	<0.0001	*0.549*	*8.3*	<0.0001*
Group 2, 9-16 births per day	**Reference**			**Reference**		
Group 3, 17-24 births per day	-0.089	-1.722	0.085	-0.083	-1.514	0.13
Group 4, 25-33 births per day	-0.068	-0.964	0.335	-0.076	-1.012	0.312

*Statistically significant.

## DISCUSSION

The COVID-19 pandemic has disrupted maternity services unevenly in Nepal. During the pandemic, medium-volume hospitals experienced the greatest average rates of declines in institutional births; while conversely low-volume hospitals experienced an increase in institutional births. Maternity services halted on average by 4.3 days, and the number of health workers per labour room in 24 hours reduced. BOR in the postnatal care units declined in all but the three hospitals that also had increases in institutional births. Our investigation on the provision and experience of childbirth during the pandemic found that women received better respectful maternity care in the lower volume hospitals as compared to the medium-high volume hospitals.

Since hospitals with fewer institutional births provided better provision and experience of care, greater consideration is needed to look at the volume of deliveries as an indication of providing respectful maternity care. The 2005 big strategy shift from home to health facilities for childbirth has been a lauded success in Nepal [42]; yet the distribution of births across facilities may be influencing respectful care. Prior to COVID-19, our study shows that some hospitals were already overburdened with large volumes of births and overcrowding, in some cases with nearly 200% BOR rates and with one health worker delivering an average of 1000 women per year. The association between overcrowding and poor provision and experience of care has been argued by others in Nepal [43] and elsewhere [44,45], and is reinforced by our study. Before the pandemic, by-passing of the primary referral health facilities (low-volume) was already a challenge in Nepal leading to overcrowding at the higher level facilities (medium-and high-volume) [32,44,46].

Our study revealed an increase in the monthly births at the low-volume hospitals (Dadeldhura and Surkhet Provincial) during the pandemic, which are first referral units as compared to their medium-volume hospitals (Bheri Provincial and Seti Provincial), which are secondary referral units. This could be an indication that women may have decided to seek care at the referral units closer to home than secondary referral units. Our study also showed that fewer women delivered in these hospitals. Studies from India have also reported reduced institutional births at tertiary care centres and a decrease in referral cases during the pandemic [5,6]. Reduced health care seeking behaviour of pregnant women was also observed in a study from Ghana during the pandemic [47]. There is an urgent need to identify where these women have given birth instead, and what sort of care these women and newborns have received. Understanding why these changes have taken place also needs to be explored. Women might have apprehension for institutional deliveries due to fears of contracting the SARS-CoV2 virus or due to lack of transportation facilities in Nepal [13], but more investigation is needed. There also remains a large gap in our knowledge around how respectful maternity care has influenced where women access maternity care.

While we observed that the respectful provision as well as experience of care was better with a decline in monthly institutional births, the coverage of some respectful maternity care indicators was still unacceptably low. Half of the women were not consented for vaginal examination; one in twenty women felt ignored during childbirth; only one in five newborns were kept skin to skin with their mothers after birth; and only half of newborns were breastfed within 30 minutes. A four-country (Asia and Africa) assessment of mistreatment during childbirth using clinical observation and follow up surveys revealed similar findings [48]. However, a three country (Africa) assessment of newborn care reported variable results for immediate skin-to-skin care (64.4% vs our 21.5%) [49]. and a multi-country study reported lower rates of breastfeeding within 30 minutes (23.9% vas our 48.4%) [50]. Provision of high-quality maternity care, including positive care experiences for women, must remain an increasing priority in global health, especially as more women [22] give birth in health facilities [51] and systems undergo redesign [33].

Our study did not support the concern that women from places reporting COVID-19 cases might receive poorer care due to stigmatization or fears of contracting the SARS-CoV2 [4]. In fact, we found the opposite. Respectful maternity care was better among women who came from COVID-19 hotspot areas, with observations showing better immediate newborn care practice, such as babies being kept in skin to skin contact with the mother and being breastfed within 1 hour. Women from these areas also reported experiencing better counselling on breast feeding, keeping the baby warm, and care-seeking for any newborn danger sign. More investigation is needed to understand why women from COVID-19 hotspots received better care.

Just prior to the COVID-19 pandemic, Roder-DeWan and colleagues called for a health system redesign of MNH services in low and middle-income countries, strengthening primary level referral facilities to provide comprehensive services, reducing overcrowding in high-level referral facilities, and providing the care needed to women closer to home [44]. The COVID-19 pandemic may actually provide an opportunity for health systems to reimagine their systems to do this as evidence from our study and others have shown reduction in institutional deliveries at higher-level facilities overall [5,7]. However, the change in the pattern of utilization of primary and secondary referral hospitals during the pandemic demonstrated in this study provides new information on the opportunity for future redesign and strengthening of services [33]. Understanding factors that influenced these changes will be key to re-building confidence in the health system after the COVID-19 pandemic.

### Limitations

This study has several limitations. None of the women admitted to the nine hospitals were tested for COVID-19, so we do not know the prevalence of the virus in this population. Another limitation is that the study was only able to examine the experience during the COVID-19 outbreak and not before. The information about women from COVID-19 hotspot areas was based on interviews, and might be subject to reporting bias. The study did not explore why women selected the facilities where they gave birth, nor did it explore women’s previous experience with the health system. The observations were only done for vaginal births, not caesarean sections. This study also has several strengths, including examining both the provision and experience of care during the COVID-19 era. Prospective data collection using trained researchers to collect information using observation checklist and semi-structured questionnaire strengthens confidence in these findings.

## CONCLUSION

The COVID-19 pandemic has had differential effects on maternity services, with a sharp decline in births at high and medium-volume hospitals but an increase in births at low-volume hospitals. This signifies marked changes in where women sought maternity care during the pandemic and more research is needed to understand these shifts. The volume of births per hospital may be an important driver for the provision and experience of respectful maternity care. There is an urgent need for a system-wide approach to address respectful maternity care in the face of continuing disruptions owing to COVID-19. The dramatic shifts in the place of institutional deliveries must be matched by service capacity to provide respectful care and so enable positive experiences for all women and newborns as well as health care workers. More research is needed to investigate the effects of the pandemic on where women give birth and their provision and experience of respectful maternity care to inform a “building-back- better” approach in post-pandemic period.

## Additional material

Online Supplementary Document
