# Supplementary material for: The perfect storm: Disruptions to institutional delivery care arising from the COVID-19 pandemic in Nepal
Source: J Glob Health. 2021 May 11;11:05010. doi: 10.7189/jogh.11.05010 (PMC8141327; doi:10.7189/jogh.11.05010)
Supplement: Online Supplementary Document [file jogh-11-05010-s001.pdf]

Supplementary file- The perfect storm: disruptions to the system for maternity care arising from the COVID-19 pandemic in Nepal

Contents

Supplementary figure 1. Conceptual framework 2

Supplementary figure 2. Study flow diagram 3

Supplementary figure 3. District distribution of COVID cases from March – August 2020........ 4

Supplementary figure 4. Provincial trends of COVID cases between March – August 2020........ 5

Supplementary figure 5. total cases by Province by sex........ 6

Supplementary figure 6. Bi-variate analysis of respectful provision of care index 7

Supplementary figure 7. Bi-variate analysis of respectful provision of care index 8

Supplementary figure 8. Bi-variate analysis of respectful experience of care index 8

Supplementary figure 9. Bi-variate analysis of respectful experience of care index 10

Supplementary table 1. Number of annual births before pandemic (2019) 11

Supplementary table 2. Mean institutional birth (SE) before (March-August 2019) and during pandemic (March-August 2020) 11

Supplementary table 3. Bed Occupancy Rate before (March-August 2019) and during pandemic (March-August 2020) 12

Supplementary figure 1. Conceptual framework

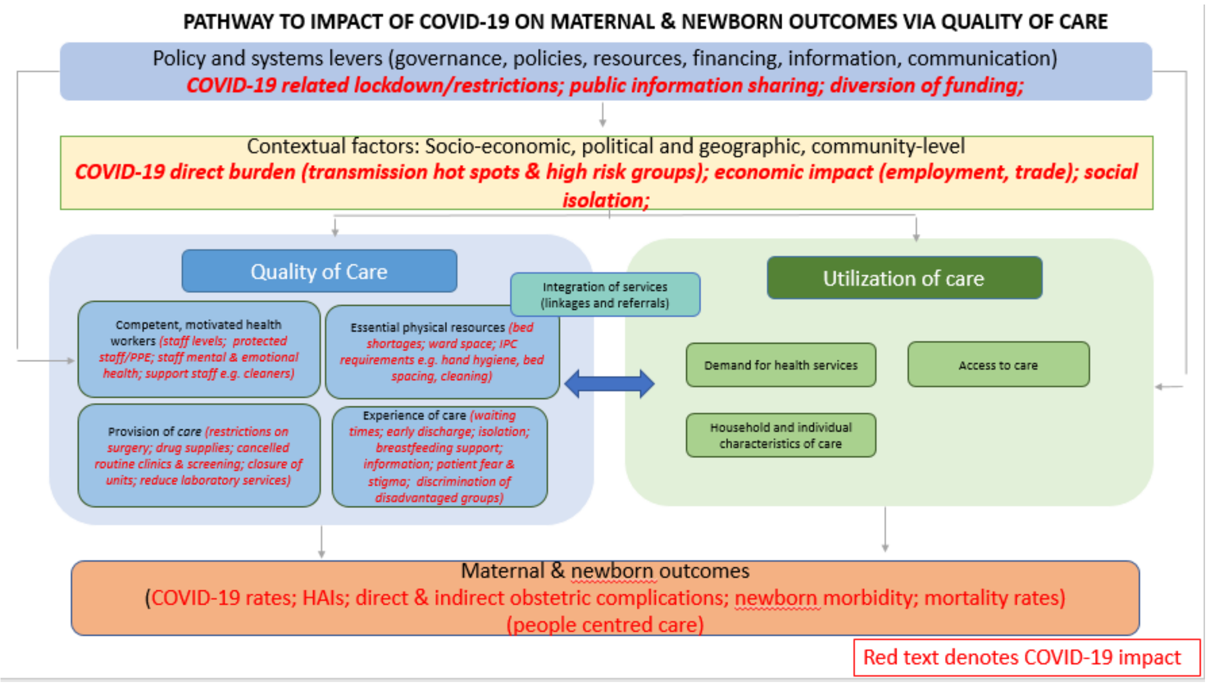

Supplementary figure 2. Study flow diagram

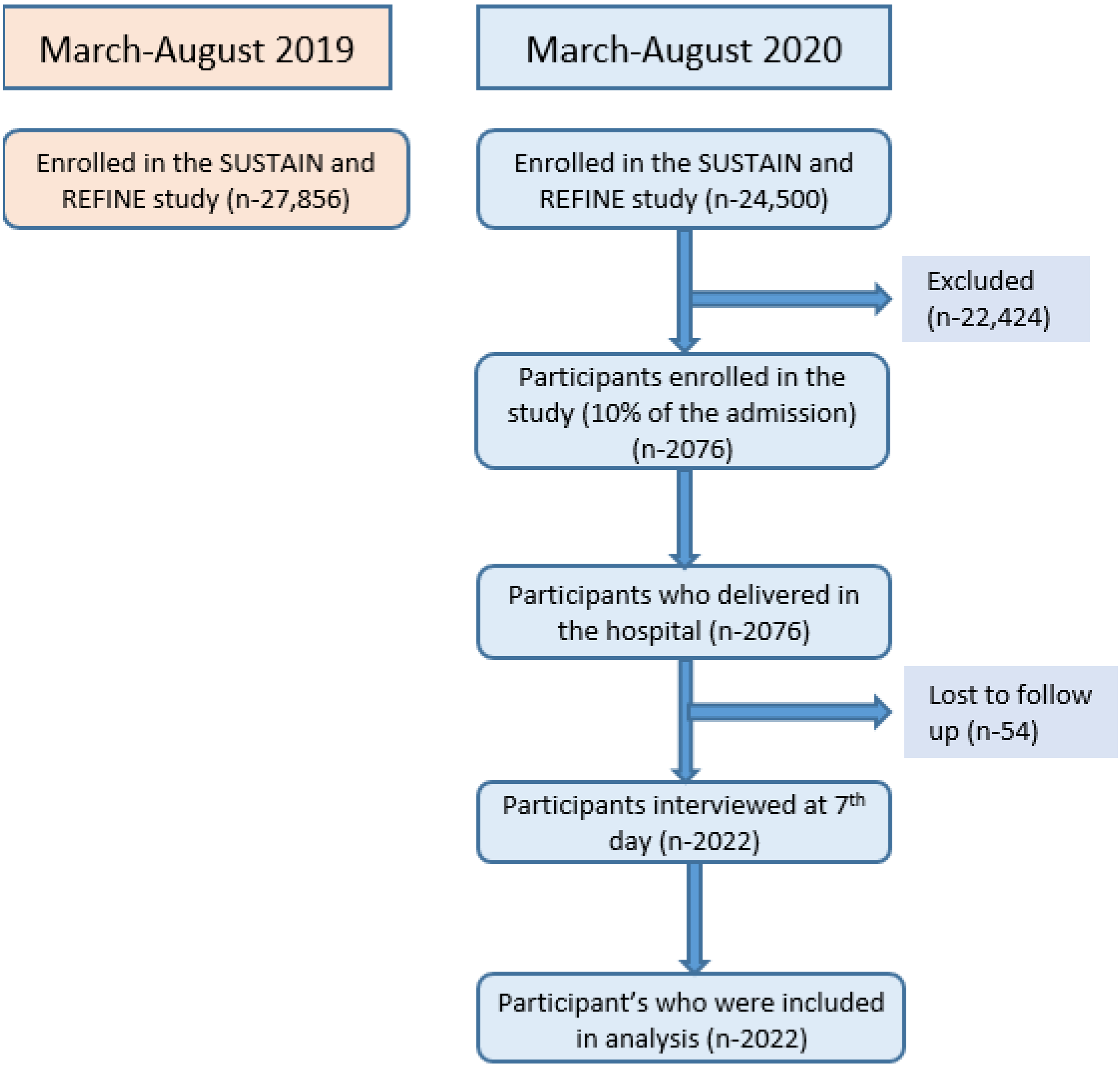

Supplementary figure 3. District distribution of COVID cases from March – August 2020

Source: https://covid19.mohp.gov.np/ [Accessed 26/11/2020]
According to the Government tracking system, there were 39459 confirmed COVID cases in Nepal between 1 March and 31 August 2020. Figure S1 shows the district distribution. Figure S2 shows the provincial trends. Province 2, Bagmati and Province 5 had the highest volume of cases.

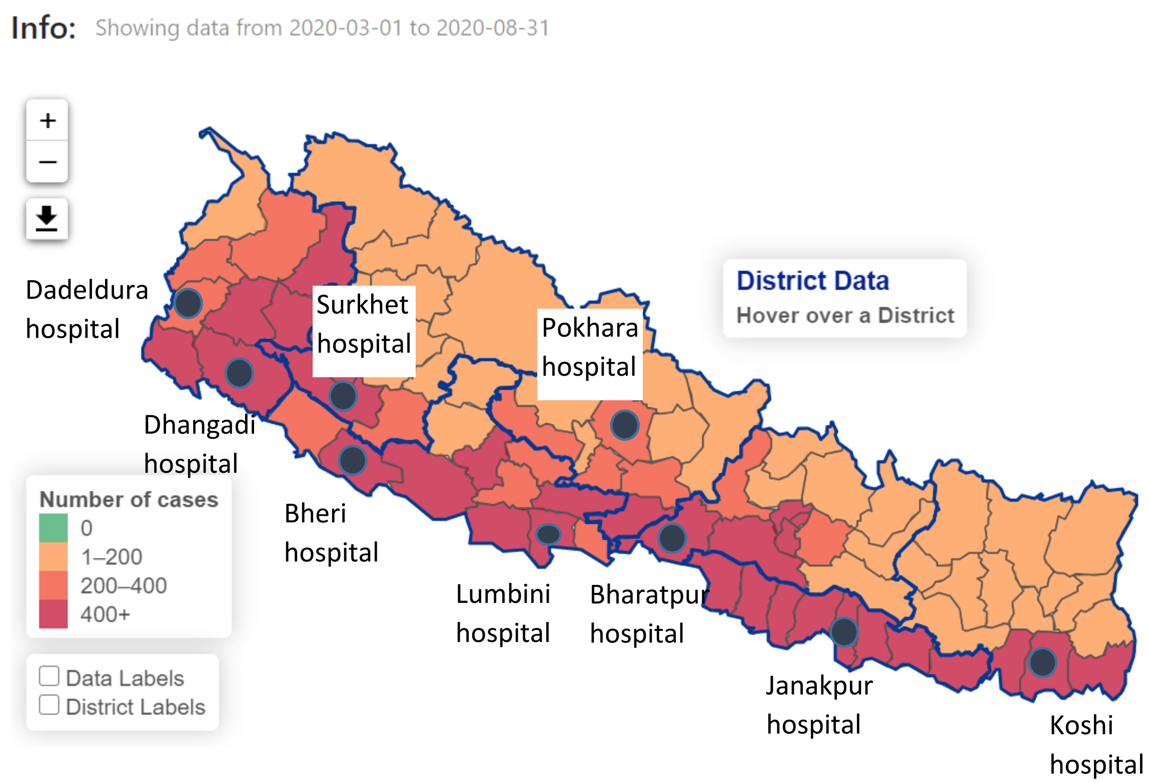

Supplementary figure 4. Provincial trends of COVID cases between March – August 2020

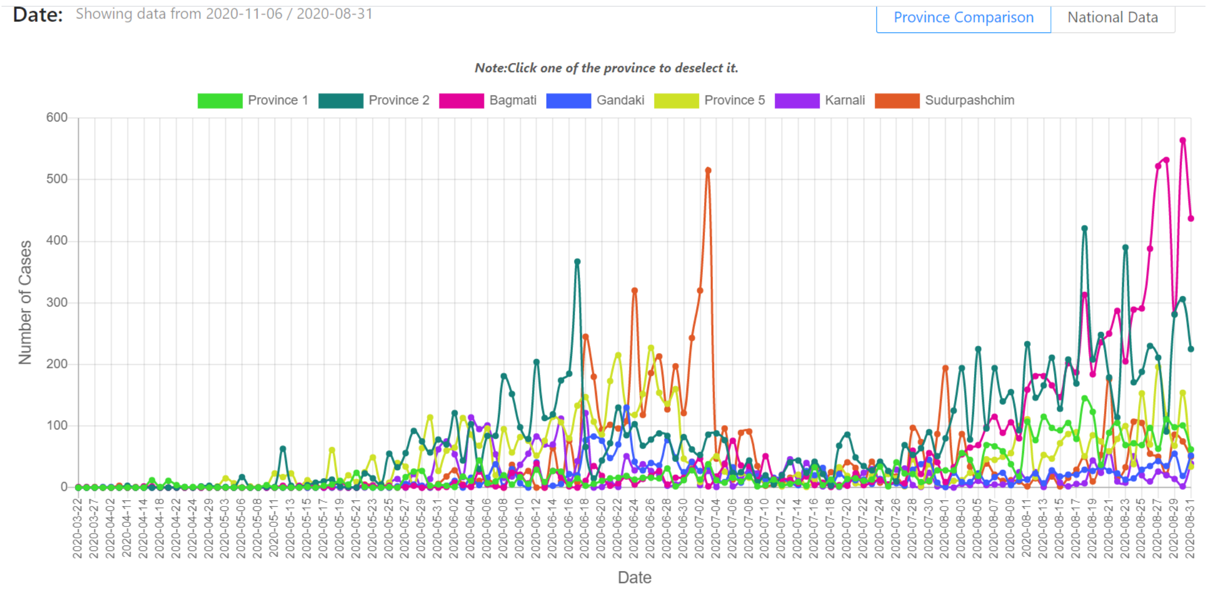

Supplementary figure 5. total cases by Province by sex

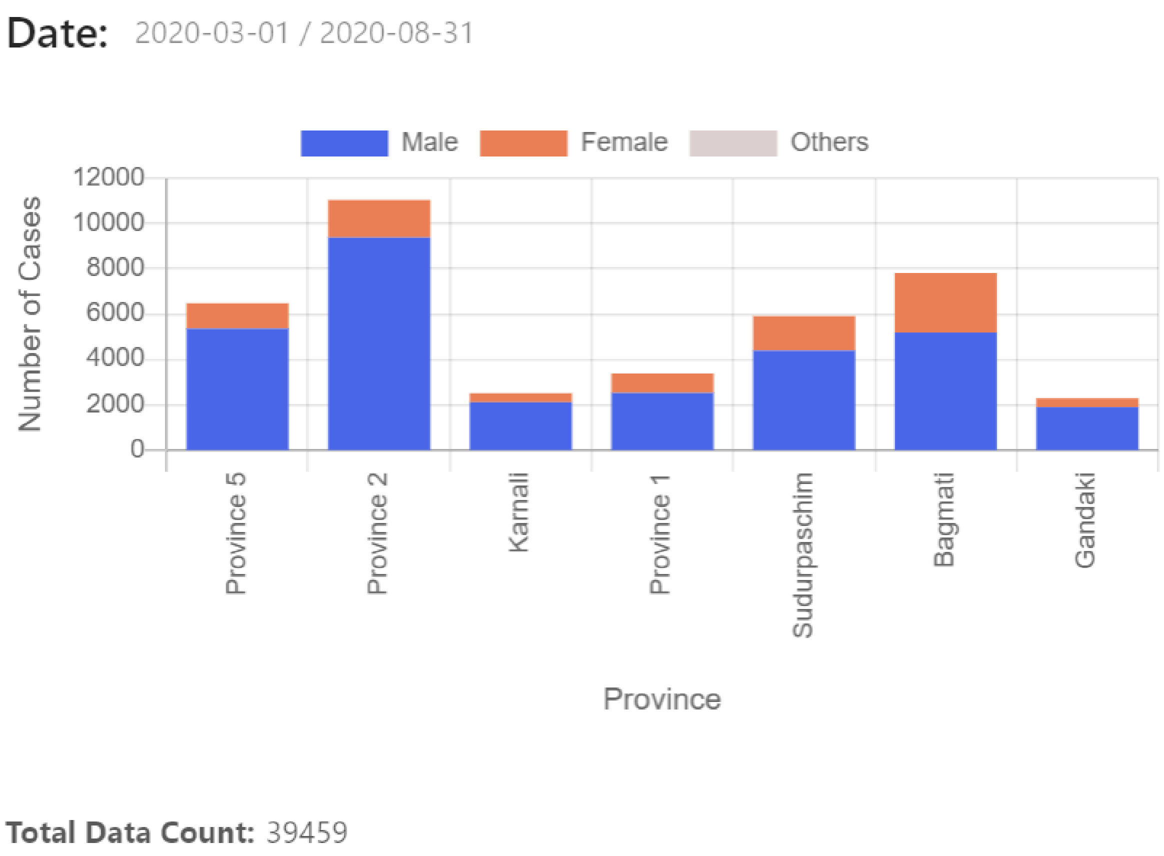

Supplementary figure 6. Bi-variate analysis of respectful provision of care index
women arriving from hotspot, ethnicity and parity

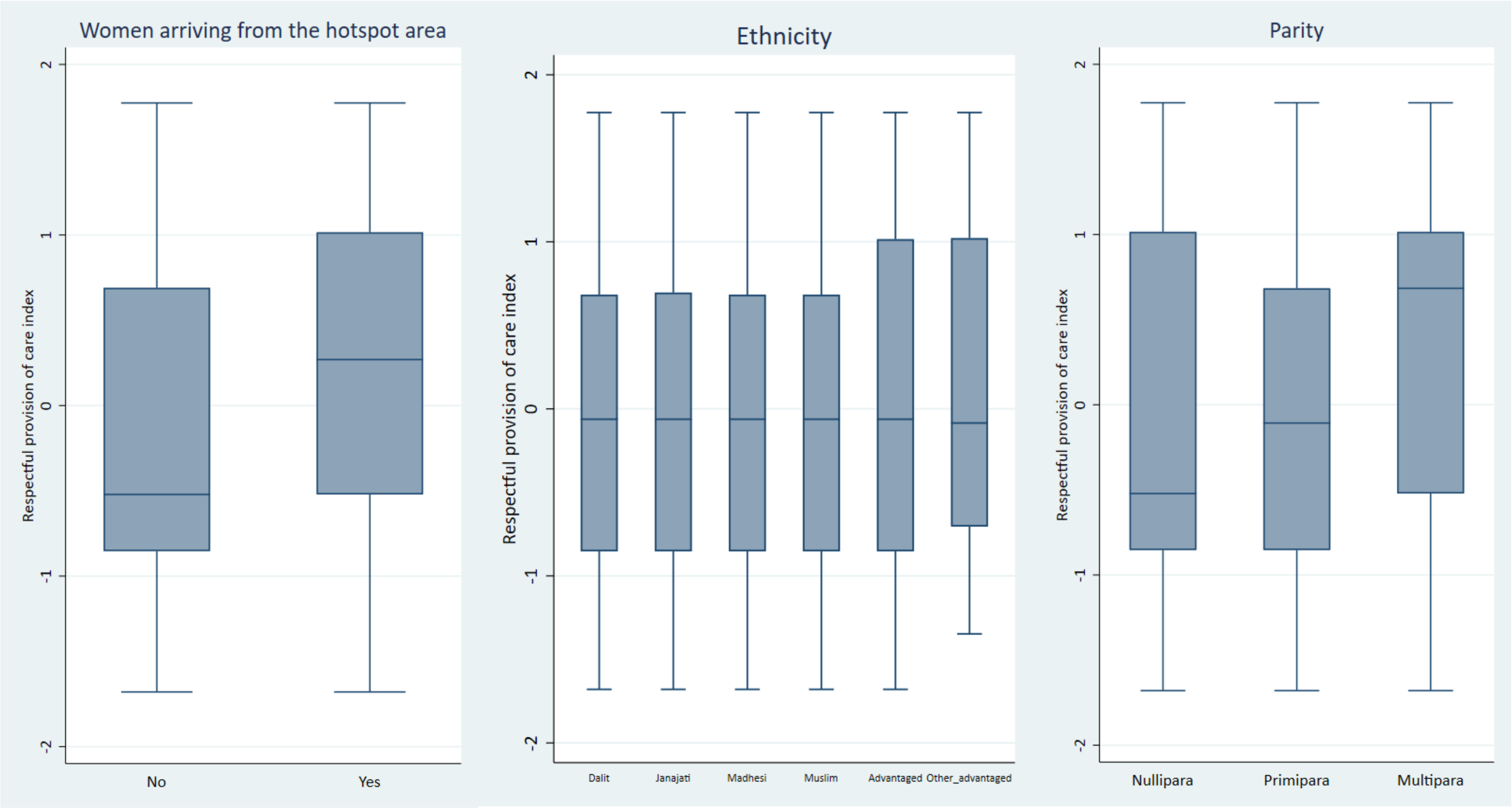

Supplementary figure 7. Bi-variate analysis of respectful provision of care index complication during admission, preterm birth and volume of births per day

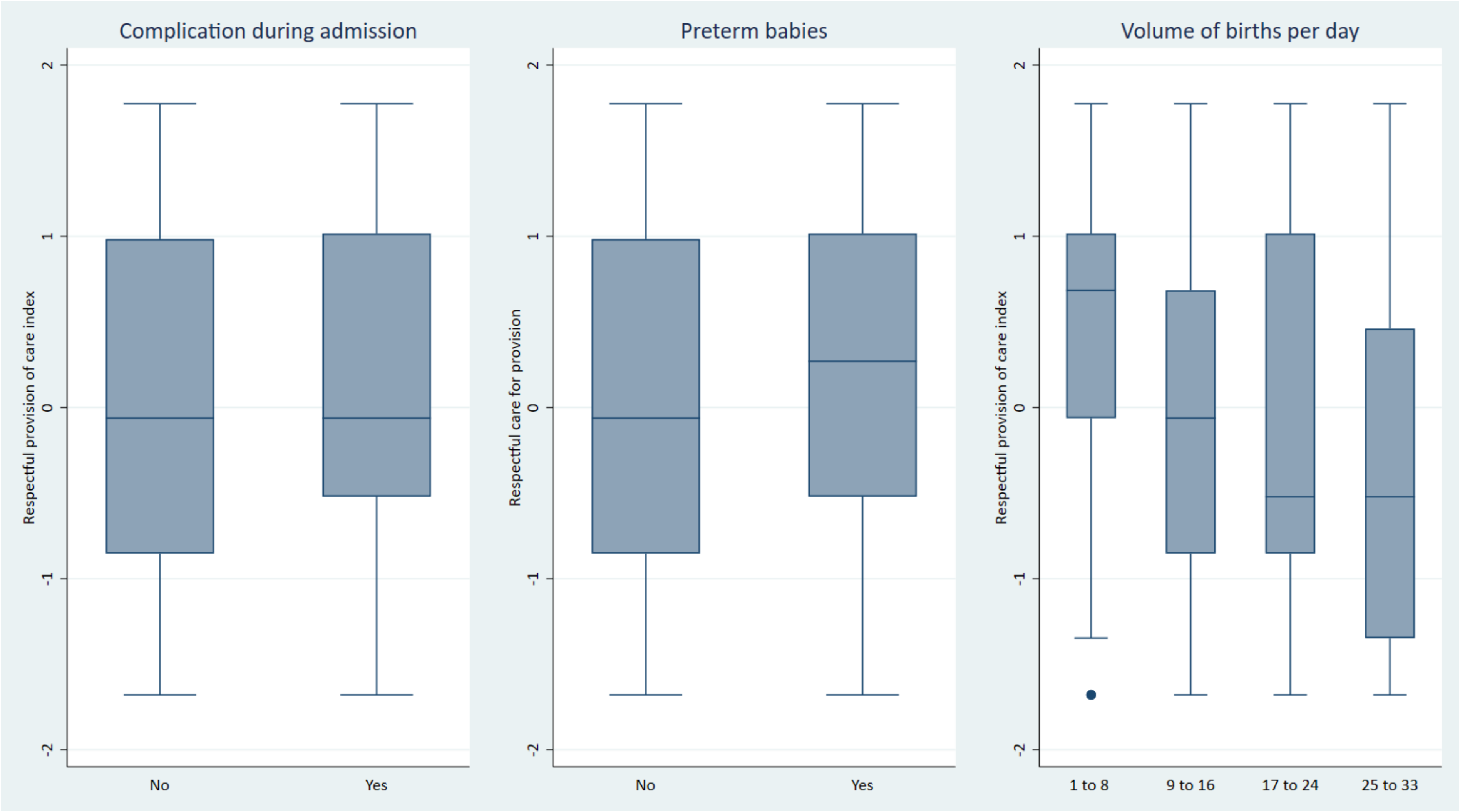

Supplementary figure 8. Bi-variate analysis of respectful experience of care index

women arriving from hotspot, ethnicity and parity

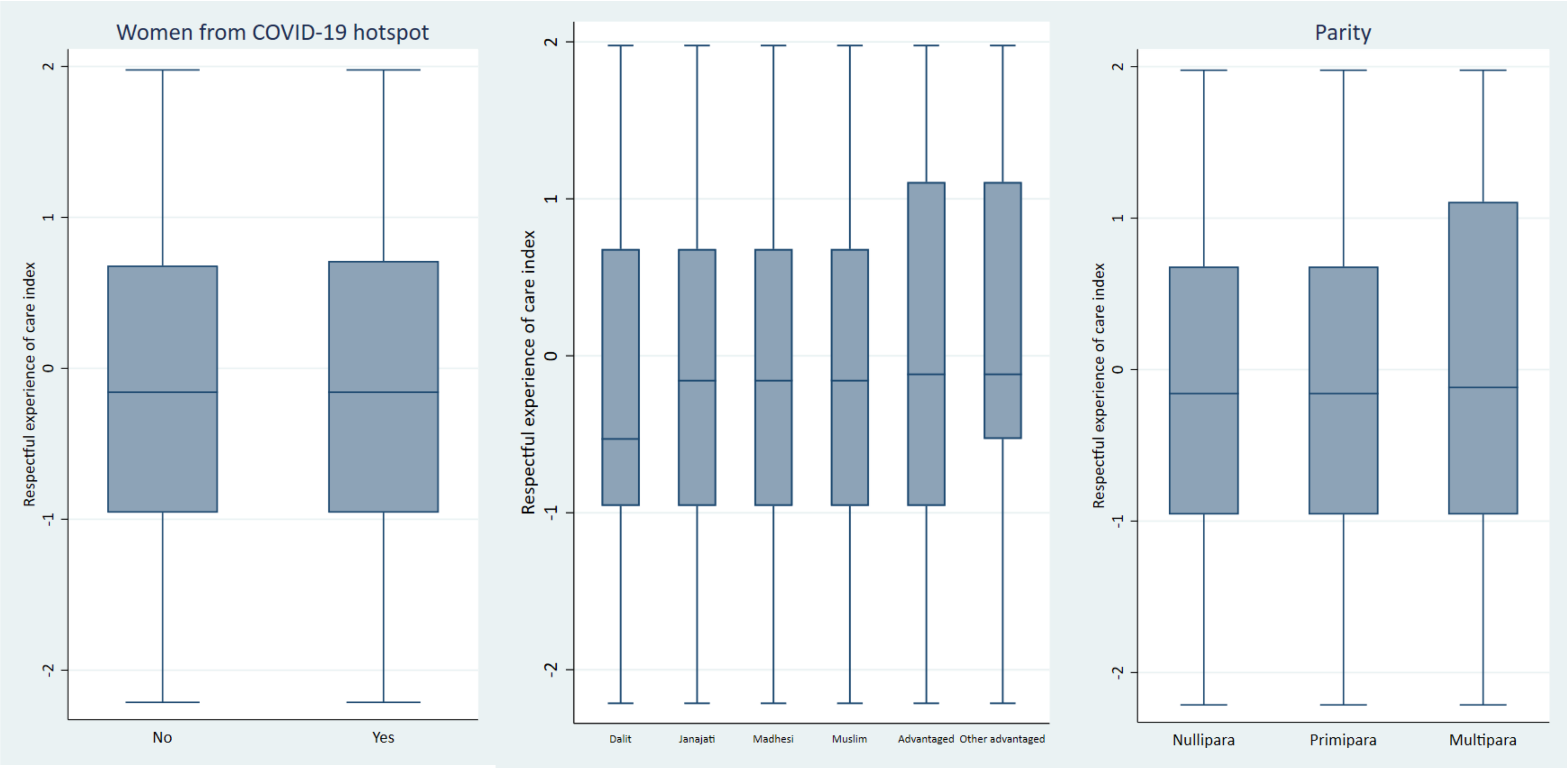

Supplementary figure 9. Bi-variate analysis of respectful experience of care index mode of birth, preterm birth and volume of birth each day

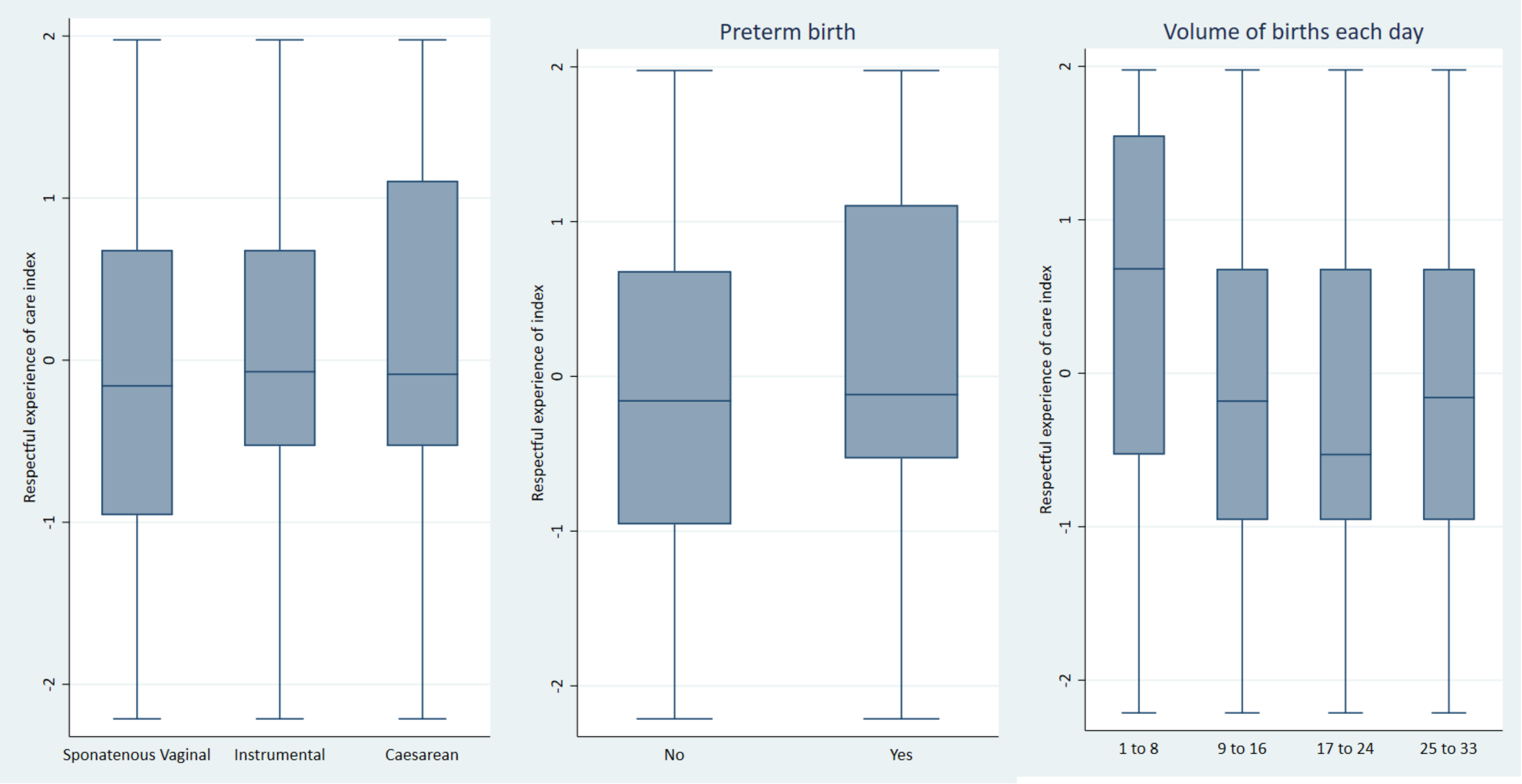

Supplementary table 1. Number of annual births before pandemic (2019)

	Monthly births (2019)	Annual births (2019)	Grouping	Annual births	Daily births
Dadeldhura	95	1140	Group 1	<4000	1-8 births per day
Surkhet	335	3961	Group 1	<4000	1-8 births per day
Bheri	373	4483	Group2	4000-6000	9-16 births per day
Seti	510	5980	Group 2	4000-6000	9-16 births per day
Koshi	528	5967	Group 2	4000-6000	9-16 births per day
Pokhara	572	6864	Group 3	6000-8000	17-24 births per day
Janakpur	565	6777	Group 3	6000-8000	17-24 births per day
Bharatpur	859	10310	Group 4	8000 or more	25-33 births per day
Lumbini	858	10296	Group 4	8000 or more	25-33 births per day

Supplementary table 2. institutional birth Count before (March-August 2019) and during pandemic (March-August 2020)

	**Before pandemic, 2019**	**During pandemic, 2020**	**p-value**	**Change**
Total	27793	24563	<0.0001	-11.6%
Month				
March	4349	4544	<0.001	4.5%
April	3552	3479	<0.001	-2.1%
May	3389	3186	<0.001	-6.0%
June	3938	3401	<0.001	-13.6%
July	5992	5363	<0.001	-10.5%
August	6573	4590	<0.001	-30.2%
Hospital wise				
Dadeldhura	582	720	<0.0001	23.7%
Surkhet	2047	2116	0.064	3.4%
Bheri	2225	1900	<0.0001	-14.6%
Seti	2963	2189	<0.0001	-26.1%
Koshi	3228	2691	<0.0001	-16.6%
Janakpur	3111	2613	<0.0001	-16.0%
Pokhara	3414	2554	<0.0001	-25.2%
Bharatpur	5175	4634	0.032	-10.5%
Lumbini	5048	5146	0.453	1.9%
Volume of birth				
Group 1, 1-8 births per day	2629	2836	0.001	7.9%
Group 2, 9-16 births per day	8416	6780	<0.0001	-19.4%
Group 3, 17-24 births per day	6525	5167	<0.0001	-20.8%
Group 4, 25-33 births per day	10223	9780	0.033	-4.3%

Supplementary table 3. Bed Occupancy Rate before (March-August 2019) and during pandemic (March-August 2020)

	**Before pandemic, (Proportion, 9% CI)**	**During pandemic, (Proportion, 9% CI)**	**p-value**
Total	91.0% (75.2, 106.8)	81.8% (65.6, 97.9)	0.421
Month			
March	94.5% (55.6, 133.4)	90.1% (49.4, 130.7)	0.74
April	78.6% (54.9, 102.4)	78.1% (43.1, 113.1)	0.431
May	67.5% (44.2, 90.7)	66.9% (37.6, 96.1)	0.978
June	80.8% (40.9, 120.8)	72.1% (33.2, 110.9)	0.76
July	108.6% (62.5, 154.6)	100.4% (48.8, 152.1)	0.335
August	115.8% (63.6, 168.0)	83.0% (36.8, 129.2)	0.078
Hospital wise			
Dadeldhura	40.6% (29.7, 51.4)	79.2% (69.5, 88.9)	<0.0001
Surkhet	68.3% (50.4, 86.1)	68.6% (56.5, 80.6)	0.976
Bheri	48.7% (37.8, 59.7)	45.2% (35.1, 55.4)	0.651
Seti	43.7% (36.4, 51.0)	25.9% (22.9, 28.9)	0.001
Koshi	69.4% (51.9, 86.9)	52.3% (40.2, 64.3)	0.141
Janakpur	117.1% (100.9, 133.3)	42.7% (27.6, 57.8)	<0.0001
Pokhara	79.4% (60.4, 98.5)	57.6% (42.6, 72.6)	0.104
Bharatpur	196.1% (144.4, 247.7)	182.7% (155.5, 209.9)	0.66
Lumbini	155.4% (122.7, 188.1)	181.6% (143.2, 220.0)	0.327
Volume of birth			
Group 1, 1-8 births per day	54.4% (41.5, 67.4)	73.9% (65.9, 81.9)	0.019
Group 2, 9-16 births per day	54.0% (45.3, 62.7)	41.1% (33.8, 48.5)	0.032
Group 3, 17-24 births per day	98.3% (81.9, 114.6)	50.1% (39.1, 61.2)	<0.0001
Group 4, 25-33 births per day	175.7% (144.2, 207.3)	182.2% (159.7, 204.6)	0.745
